# ‘Islamic fatalism’: life and suffering among Bangladeshi psychiatric patients and their families in London – an interview study 2

**DOI:** 10.1080/13648470.2013.853598

**Published:** 2013-11-15

**Authors:** Roland Littlewood, Simon Dein

**Affiliations:** Departments of Anthropology and Mental Health, University College London, Gower Street, London WC1E 6BT, UK

**Keywords:** Bangladeshis, London, fatalism, predestination, God, Islam

## Abstract

An interview study of 44 Bangladeshi patients and relatives in eastern London demonstrated frequent appeals to God and deprecation of personal agency. This paper offers an interpretation of this apparent ‘fatalism’, which argues for the logical downplaying of human agency and ambition in archaic Arabia, contemporary rural Sylhet and among first generation Sylheti migrants in London.

## Introduction

Islamic societies have been characteristically identified by Europeans as ‘fatalistic’ – that is, as having a resigned attitude to human action and misfortune through placing responsibility onto fate (or rather onto God).

The starting point for the current paper was an interview study (Littlewood and Dein 2013a, 2013b) of how one particular Muslim group – Bangladeshis in Britain, both born in Bangladesh and their British-born children – experiences states characterised by biomedicine as mental illness, how they understand and deal with these states, and their satisfaction or otherwise with the government health services provided. Whilst not strictly ethnographic, the paper places particular emphasis on respondents’ own statements. The social and economic conditions of London Bangladeshis are briefly summarised in Gardner (1995, 2002) and Dein (2009), and the social anthropology of mental illness in Sylhet by Callan (2005).

There are approximately a third of a million immigrants in Britain from Bangladesh, 95% of them from the north-eastern district of Sylhet, an area with a landholding peasantry rather than being comprised of the large estates found elsewhere in East Bengal (Gardner 1995). The earliest migrants were seamen who settled in London, acting as brokers in the 1960s for their relatives by obtaining employment vouchers for factory work. With the cessation of Commonwealth primary migration, entry has since been restricted to secondary migrants (family members). Relative to other South Asians in Britain, the families reunited rather later on and are the ‘last’ South Asians to settle (Gardner 1995, 117), with more recent emigrants from Bangladesh going to Saudi Arabia and the Gulf, but they cannot obtain permanent residence there. Bangladesh is a low-lying country, a third of which is susceptible to unpredictable flooding each year: and it is rated as one of the ‘least developed’ nations on the Human Development Index (Colwell et al. 2003, 105). Sylhetis are largely Sufi-influenced Sunni Muslims (but 10% are Hindu) who follow an Islamic path that is depicted by ‘purist’ (clerical, scripturalist) Islam as contaminated with Hinduism in its reverence for, if not actual worship of, saintly *pirs*, a more thaumaturgical approach to the problems of social and religious life, and with local practices of oblations, music and dancing. Wealthier Sylhetis are able to practice a more ‘purist’ Islam – female seclusion and ritualised formal religious observance – whilst at the same time pursuing religious and/or secular education (Gardner 1995).^1^ Marriages and kinship patterns are traditionally through male-descended lineages with a preference for arranged patrilateral parallel cousin marriage, and continuous generational alternation between joint (parents, sons, son's wives and children) and nuclear households. On the death of a father, the land is equally divided between the sons (with daughters receiving a half-share), and until recently women were restricted to a household role. In Britain, Sylhetis have settled particularly in the East End of London where the men work largely in ‘Indian’ restaurants and garment factories. In the sample for this paper, only two people cited an occupation that was not restaurant based. Bangladeshis are generally considered to be the poorest and most socially marginalised minority community in Britain (Gardner 2002; Eade and Garbin 2002); and probably the most self-effacing.

The 25 identified patients in the sample (16 male, nine female) and the 19 carers (mostly relatives, three male, 16 female) were interviewed for upwards of an hour each by one of two Sylheti-speaking Bangladeshi professional community workers, both women, who had previously completed an MSc with the authors in medical anthropology and health (see the Acknowledgements section). One woman interviewed was both a registered patient and also a carer for her brother, and some of the other carers were receiving counselling or other psychological support. The ages of the patients ranged from 65 years to 27, of carers 73 to 18. A clear majority of the total sample were born in Bangladesh, 40 out of 44, but two patients and two carers were born in the United Kingdom. All, both British and Bangladesh-born, had some first degree relatives living in Bangladeshi. To try to tease out any local differences in interpretation of what biomedicine would term physical and psychological illness, the sample included patients with physical illness (seven instances) and carers for eight patients with physical problems or somatic symptoms as well as purely ‘psychological’ illness (29 instances, including two both physical and psychological). Most informants had some schooling with an average of 6.2 years, two had none (one, ‘Almost nothing – I did go to school for a while’), three had an undergraduate degree or diploma, one an MSc. In three cases, the educational level was uncertain. All respondents were Sunni Muslims and at home spoke Sylheti, the language for all but two of the interviews. About half the women respondents also spoke some English; the others used their children for translation when outside the home, dealing with hospitals, schools or the local council officials.

A convenience sample, informants were found by random at two mental health day centres and one social services support centre. They were interviewed privately in Sylheti following a loosely structured schedule; all interviews were recorded and then transcribed into English; in three cases the audiotaped interview was succeeded by a lengthy conversation, which was written up concurrently. No one contacted refused participation or recording. Respondents were each paid a modest sum of £20. Quotations here are from the English transcriptions subsequently read by the authors and agreed with the two interviewers. In this paper, the authors have not attempted to correlate the hospital diagnosis with the local understandings, so the responses are just a general account of what might be called ‘distress’ among patients and families: this is justified by the frequency of change of medical diagnosis and the respondents’ own uncertainty about specific causative or aetiology. So, the respondents include a mixture of people that a psychiatrist would define as psychotic, as simply distressed physically or psychologically, or as quite well.

Examination of the transcripts and discussion with the interviewers revealed a high proportion of immediate explanations for misfortune involving *jiin* (spirits) and *jadu* and *korni* (sorcery); frequent sorcery accusations against relatives, generally in-laws but sometimes against more distant sponsors acting in concert with the in-laws; the ambiguity of *tabij* (amulets) which could harm as well as heal, although their use seems to have been near universal; an ambivalence about Islamic clergy and religious figures both in Britain and Bangladesh who were often felt to have their own material interests principally at heart; and a high level of trust in psychiatric doctors, although informants accepted that some ‘spiritual illnesses’ would not be affected by biomedicine (Littlewood and Dein 2013a, 2013b). The authors considered from their wider accounts of everyday life and work that this population, relative to other minority ethnic groups (and perhaps to other Bangladeshis), was comparatively isolated from mainstream British society (see also Eade et al 1996). The current paper concentrates on the distal causality and providence associated with their references to Allah (God).

## God

Most respondents when talking of proposed action or anticipated good fortune, would briefly insert *inshallah* (God willing): ‘next year I will go to Bangladesh *inshallah’*; ‘I hope my children don't get this sickness: hopefully Allah will keep them well’; ‘I am hoping to get my daughter married soon, Allah willing’. (As for White Britons, such an invocation was only for desired events, although theologically it would make sense for anticipated adverse events: so, no ‘God willing, I will have no children’.) Allah is the ultimate explanation for any event, even if you yourself strive: ‘My husband gets appointments to see the psychiatrist every six months, if it gets worse they increase the dose of the medicine and tell us what to do. The rest is up to Allah.’ *Inshallah* is less an affirmation of a particular desire or endeavour than a wish that that desire will be within God's will: ‘Do not say of anything: “I will do it tomorrow” without adding: “If Allah wills”’ (Qur'an 18: 23, The Cave). And to Him the credit: ‘So one of our informants – by the grace of God three of my children have graduated. I am grateful to Allah that my children have achieved so much’ (in English).

And Allah can be supplicated, possibly for a good life: ‘So I prayed, I request Allah to heal me, to grant me a long life, to enable me to earn money, to keep me well, to forgive me for my mistakes, and to take me upon the straight path [towards God], that's what I used to pray’. Or else for actual healing: ‘I have no physical strength [he had had a stroke], and I cry out to God for all this suffering; rather than give me all this suffering … I mean when I am lying in bed, I am begging my wife, “please help me get up, please help me get up”; but, you know, it's hard for her too. So I constantly think about this, and I implore God to help me. A burden: do you think anyone likes carrying a burden? It's heavy, and you won't like carrying it. So, I am like a heavy burden for others in my family. I pray to God that God doesn't make anyone suffer like me.’

Or if God is not solicited to actually heal, then more modestly he may be asked for the strength to bear a problem which in His wisdom he has chosen to inflict. ‘Oh God, what should I do, I am in such hardship, this person [her husband the patient] is giving me so much grief.’ ‘I always have faith in Allah: “Whatever you do Allah, please give me the strength to bear it so I am not a burden to my children”.’ Virtually all the patients and carers recommended fortitude and faith in God: ‘It says in the Qur'an that if you pray with faith, then it will help. I feel that my worries do lessen with prayers. Sometimes the GP gives me some medicine which has no effect whatsoever, and I cry out to God from my heart and pray to be helped and healed; he can choose to take me or heal me. When I say these words, I find comfort and relief, and that then seems to make me much better. I leave all my trust upon God to take care of me. Prayers seem to bring ease. It's actually what can give peace of mind.’

And ‘If I found a good spiritual healer, I could give it a try. But I have not found any. I say prayers to God; I also send salutations and blessings to the Prophet [with specific prayers for this] and then I blow over my chest: I say “*la ilaha illāllah”* [There is no God but Allah]. And I say that God sent me to this world to go through this.’ And Allah will often warn you although not by explicit communication (cf. Dein and Littlewood 2007). ‘I think, it was Prophet Muhammad, or maybe a saint [in a dream]! Someone like that, maybe I am walking on the wrong path [not acting in accordance with religious teaching], and so I was shown [this] as a reminder to come back on the straight path. Maybe it's a message: you have a future to live, you have children to bring up properly, your children might lose their way on the path, if you don't try and return on the right path.’ If not actually a direct communication with Allah then ‘sometimes I sit down to read the Qur'an [but] then I pray to Allah with the *tasbih* [prayer beads]. And music gives me some comfort, some peace.’

Although all sickness ultimately comes from God as a test, as a punishment or as simply something mysterious, some problems are seen as more immediately naturalistic (including from everyday life) or else caused by human sorcery: ‘If I see that it's a kind of illness that mullahs treat, then I will suggest to them [her neighbours] to go and consult a mullah.’ ‘I have been to so many doctors and they tell me it originates from worries and the rest is perhaps from God’, says a patient's wife. One woman with a ‘depressed’ (doctor's diagnosis) husband said: ‘Nothing bad happened because of me. People say that somebody has done *jadu*^2^ or that a ghost or other spirits have influenced him. He became ill all of a sudden you see. I cannot say for sure whether it was caused by worries or was from God or caused by any jadu.’

‘This pain is giving me a great deal of suffering, everything else does too but this pain gives the most. What do I do? I sit quietly and call upon God. Sometimes I think that I should go and see somebody for a *tabij*^3^ or something but then I wonder, well – what would be the point? My illnesses are nothing to do with *ufri*’.^4^

And ‘Someone from Bangladesh did it [the spirit affliction] to get more [money] from him … If this comes from Allah then I will be patient. If it comes from something else, then Allah will judge this.’ This distinction between spiritual and immediate magical or physical causation is often made when one sort of treatment is effective or unavailing:

‘Well, if the Satan^5^ is going to do something [to cause the symptoms], or whether it is something due to me [from God, for his actions], there are cures for both of these. For example, if you went to bed, and had breathing difficulties as a result of Satan having attacked you and holding you down, then, you can recite prayers from the Qur'an, and then it will leave you – you will have healing. So, if you want to successfully liberate yourself from that, you will have to be *pak.*^6^ You will have to go to bed in a pure state, then of course, none of these entities can come near you; that's according to our religious perspectives and beliefs, but it's not something adhered to or believed by people in this country. How can they, they are not Muslims, they do not believe in the Qur'an.’

But many remain uncertain, arguing that only Allah knows the immediate cause or the real spiritual status of the healer. ‘I cannot say for sure whether it is caused by worries or was from God or caused by any jadu.’ ‘I cannot blame someone I have not seen, I think it is God's will.’

The *pir*^7^ said something has possessed him [her husband]. You know they say these kinds of things in Bangladesh. Whether it is true or not, only God knows. I don't know. His parents got the *tabij* and things and visited different pirs’ places, used blessed water and oil things like that. They did this before and after our marriage. Before giving the tabij the guy sits in a trance. Only God know and he knows: I don't know what he does. Whether he is conscious or unconscious I don't know. After this he gives the tabij. Then tells you to form the intention of sacrificing a cow or something for Allah, so you do; He does his healing. You then see some good [healers] and then some bad, only God knows. For these few years no one is bothered to help. It doesn't improve. We have given so much, given money and the cow. Whenever he went to Bangladesh, they did this.

Sometimes it becomes difficult to adjust proximal against ultimate (divine) causality in a particular instance, but if a presumed useful treatment does not work, ‘I think it wasn't Allah's will [that the tabij would be effective]; if it was, then just by sitting here he would get better. If there is no Allah's will, then nothing would help.’ Even proximal treatments cannot work without ultimate divine sanction.

Spiritual purification and prayer whilst recommended as remedies for illness can also be used to be effective against unpleasant thoughts:

I recite prayers from the Qur'an and other Islamic books that I have learnt, and I ask God to help me, and sometimes I do receive help from God when I am frightened. I do receive help. It used to happen to me about six months ago, I used to feel quite scared, like someone is trying to capture me, kill me, but I came out of it, God helped me. For example, at night I see that someone is trying to suffocate me to death. Then I will purify myself with *wudu* [ritual ablutions], and will recite Ayatul Qursi [the Verses of the Throne, from the Qur'an] seven times and blow over my chest, and then I see that nothing tries to suffocate me during that night. It is stated in the Qur'an that efficacy of these prayers depends on one's state of soul.

Thus, for some, amulets and other spiritual aids can only work if you are spiritually prepared. ‘If someone's heart doesn't want religious help, it's no use giving them grief’ comments another, ‘Using tabij and things is not useful. I read the Qur'an before I go to sleep and do my prayers. There is nothing else really.’ And three people were totally opposed to the clergy's amulets: ‘I don't like going to see a mullah and paying them twenty to thirty pounds. I just cry aloud to Allah.’ Another said:

I don't need it, there is one God. If you do it yourself, there is no need for the tabij. Using the tabij is not right. Before, there were the righteous mullahs who said give money to the mosques. The ones who give the tabij now, they want money for it. Yes, that's how it seems, sister. I don't believe in these other forms of treatments. It's not good to go near these kinds of treatments. I don't think it is a good idea. They are bad people [who offer tabij], they make things worse for people.

These three, among the better off and educated, think an amulet is rather too much like ‘magic’. The one (British-born) university graduate totally objected to the very idea of amulets:

basically, if you are a true Muslim and you really believe that Allah can help you, then you make *du'a* [prayer] for Allah to help you. If you have something on you, even if it's the words of Allah, and you put your trust in that thing … I can't. You know I need to learn from the Qur'an, I need to teach myself things, you know like morals, and how to be a good Muslim and a good person, and how to treat others well. Not to lie and stuff like that – but if I want help it would be by asking Allah, not by putting my trust in things – because that's like idol worship. If somebody wears a ‘tabij’ I don't think they have faith in God. Some people might get confused. If they put their faith in that thing then that's completely contradicting everything that they're meant to believe in.

But for most, effective healing involves practical measures, medical or spiritual, and also God's immediate support, both together. One person with apparently psychotic experiences said:

Actually, you know, I have female fairies^8^ that are kind of with me, they are always near by, and I am always chasing them! But since putting the tabij on, they cannot come near me. That's what the *measaab* [student at a madrasha] has said. You know, it's like all they do, is play with me, and I am playing with them. But I can't catch them and they can't harm me, because I have God's shade upon me, he protects me, otherwise they would have killed me.

And many are confident that God always answers prayers in some way and in some form:

Last week a lady came and requested me to give her some blessed water, and I instructed her as to how she should purify herself, and how she needs to go to bed in a purified state and sleep on her right side, and she should present her request to God. I told her that I had no power to do anything, it was God who has power: she should request God with prayers, and God shall respond to her prayers; might take days, might take a year or more for God to respond; but he does respond to prayers. Muslims believe that God does respond to prayers, we Muslims believe in that – we have that faith. The medicine from the doctors seem to offer an immediate relief, and the other things: well, they are blessed by God, so it takes time, but if you have faith in it, it will work. It works slowly…

If you were to have a stomach ache then if you take recited water^9^, Allah would pardon you [and relieve the symptoms]. But I don't know what he [her husband] has. What are you going to tell the mullah, what can they give him? Not everyone can understand his illness. Some people in this country [Britain] don't like the tabij. We should have the belief that Allah will help, even if you are mad, you have some faith Allah will help. Medication helps, that's how the world is running. The Qur'anic stuff [tabij and pora pani], whether it helps or not, it would not have any bad effect. Nothing bad will happen, it will stay good. Even if it doesn't help, it wouldn't make it worse. Medication can either make it worse or better.

But some think amulets can be harmful or have almost given up hope: ‘So, I am always grieving about this, worry about this, that perhaps I have sinned. Will God return my physical strength? Will he let me live longer? Will he return my strength? If not, then, will he take me? I think that's better than others having to endure me.’ Or as a patient's relative admitted to praying, ‘Oh God please take this person away, we cannot cope, he is draining us.’

Ultimately God is responsible and He responds to one's deeds:

If it is God's will, maybe the sickness might be passed on [to other family members]. It's a short life. If you do good things, it's good. If you do bad things, sometimes you get a punishment here, and some in the hereafter. And for worries, you say to yourself that all that is happening to me is happening due to God's will, so what's the point in worrying about these things? For example, I have a mother and she died. I cried but I consoled myself that she has been taken by God to him, and that belief was reassuring and it helped me in my grieving. When my daughter-in-law passed away, I was feeling so upset and anxious, and then I said to myself: ‘well we will all have to die one day.’ And that brought me a sense of relief. These matters are in God's hands, that's what we believe as Muslims. The length of our lives, the level of our sustenance, and the time of our death: these are matters decided by God.

And: ‘I believe in Allah and he has given me this test, and if I do take it easy [stop worrying] I'll be the gainer. So I have to ask Him for forgiveness if I did anything wrong in the past. Why is this happening? Maybe we did wrong that Allah is not happy with. I'm getting punishment so I need to change: to do more reading and praying, try to pray five times a day at exactly the right time.’ ‘I always think about these things and cry to God I don't know what sins I have committed.’ A mother with a psychotic son: ‘I took him back to Bangladesh thinking that he might get some peace. But it didn't work. Here [in London], getting medication and things, gradually Allah has pardoned [relieved] him.’ And if the illness is clearly willed by God, that can to an extent displace individual agency and thus perhaps stigma: ‘His father was such an intelligent and well-known person: everybody understood this happened because of Allah's will, so why should our reputation or our name suffer?’

## ‘Islamic fatalism’

There are thus limits to human agency in Islam: and it seems that God is particularly appealed to in the instances of the more chronic, the more serious and the less intelligible. Whatever happens, it is God's will, although He may be responding to one's past actions or else be simply inscrutable. And there is a long European tradition of ascribing to Muslims *fatalism*, of not merely accepting God's ultimate providence but of giving up too soon, of not pursuing actions such as healing for long enough (by European measures) but passing the call for action onto God, a critique which Edward Said (1978) has argued supported colonialism. A good example is provided by the Arabist and traveller Charles Doughty in the 1880s when he attempted to offer some sick Arab children medicine but was told their fate was in the hands of God. He reported: ‘If I said, “Are not the medicines the gift of Ullah?” They answered, “We are trusting in Ullah”… This is the supine nature of Arabs, that negligent of themselves and expectations of heaven to do all for them, which they take for a pious acquiescence in the true faith: this fond humour passed into their religion we have named the *fatalism* of the Mohammedians’ (Doughty 1888, 155). All Bedouin are ‘improvident’ says the modern English adventurer Wilfred Thesiger (2007, 113). In a sense then this fatalism is taken as particularly Arab (and indeed pre-Islamic): ‘this fond humour *passed* into their religion’ [our emphasis]. T.E. Lawrence blamed the desert for ‘this climatic Arab God’: ‘It was easily felt as an influence, and those who went into the desert long enough to forget its open spaces and its emptiness were inevitably thrust upon God as the only refuge and rhythm of being’(Lawrence 1926, 29).

Something akin to this was found among the pre-Islamic Arabs as *dahr*, a notion of Fate, which appears very similar to that of classical Greece (Bowering 1997). *Dahr* was modified by Islam in that a personified deity now controlled fate. What Allah has determined happens (Bowering 1997: 58); when you are still in the womb an angel writes down your daily life, happiness and illness, hour of death. ‘Unavoidable as fate and irreversible as time, each instant happens solely through God's very own action’ (Bowering 1997: 58). And each moment of time is God's creation. The Muslim historian Ibn Khaldûn (2005) in the fourteenth century CE presents the desert as the source of group solidarity, spirituality and creativity, successive movements that establish urban dynasties which eventually become decadent and decay. The frugal inhabitants of the desert are found to be more religious and more ready for divine worship (Ibn Khaldûn 2005: 67). Often cited for his rationalist explanation of social change, Ibn Khaldûn however sprinkles his text with comments such as ‘God gives success where he wills’, ‘This is how God proceeds’, ‘According to the way He has planned’, particularly when talking of major dynastic and cultural changes, whereas the smaller changes in history are understood through individual human psychology and self-determination: ‘The final outcome is up to God’ (Ibn Khaldûn 2005: 24). (Compare structural functionalism and agency in social science.) This seems similar to the earlier view of Abu'l-Hudhail (ninth century CE) that man can be said to be the agent only of acts he can contemplate; the rest is up to God (cf. Fakhry 2004, 49–50).

Europeans can be quite scathing about such apparent fatalism which they have characterised as ‘defeatism’: ‘As far as the Arab personality is concerned, there can be no doubt that the same belief in predestination or fate … exerts considerable formative influence. It engenders an attitude of passivity and of disinclination to undertake efforts to change or improve things. It especially discourages long term efforts which require advance planning, because any such activity might come dangerously close to rebelling against Allah, and his will as manifested in the existing order of things’ (Patai 1983, 163). So, to quote one Western scholar, as their new religion passed beyond the Arabs to become a wider religious community, ‘It has become a commonplace that Islam is a fatalistic religion which teaches that everything is determined in advance and that man is unable to do anything about it’ (Ringgren 1967, 52).

In considering the validity of this idea, we first need to clarify the distinction between this attribution of defeatist fatalism (e.g. Senior et al. 1999) and the doctrine of predestination – what is written on ones forehead – (*qada at qadar*, lit. judge of judges) which is certainly Qur'anic: ‘For if Allah should touch you with adversity, there is no remove of it except Him; and if He intends for your good, then there is no repeller of His bounty’ (20: 107–109). ‘The fate of each man we have bound about his neck’ (17: 15); ‘Nothing will befall us except what Allah has ordained’ (9: 51). There is some conflict between surrendering to God's will and the search for treatment at times of illness. The Prophet was supposedly asked ‘Do our supplication, medication and methods of prevention prevent anything that God has willed?’ He replied ‘These are also part of God's will.’ Although illness occurs according to God's will, every Muslim is commanded to protect himself against it, deploying preventive methods (which also work by God's will). The Prophet approved the principle of medical treatment and encouraged his followers to seek it. In a *hadith*, he is quoted as saying: ‘Make use of medical treatment, for Allah has not made a disease without appointing a remedy for it, with the exception of one disease [whose] name was old age’ (Sunan Abi Dawood, 28: 3846). And in another: ‘There is no disease that Allah has created, except that He also has created its treatment’ (Sahih al-Bukhari, 7:71:582).

Reconciling predestination (by an omniscient, omnipotent but inscrutable God) with free will and human action was a similar problem for Christianity (Romans 8: 28–30), and came to the doctrinal fore with St. Augustine, and later in Calvinism, which solved it by arguing that although you could never be sure of being ultimately saved, you had to act as if you were, and if God favoured you materially in this life then that was a promising sign. Unlike Christianity, Islam has no doctrine of original sin (Conrad 1999); without going too deep into the theology of Islamic predestination and free will,^10^ simply God has inscrutably decided who is to be ultimately saved or damned, and He also decides whom to punish in this life. (‘If Allah should touch you with adversity, then there is no remover of it except Him’: Qur'an 20: 104). Yet at the same time as one is free to act, you are obviously in with a better practical chance if you choose to follow Islam however matters are ultimately determined (‘And had your Lord willed, all those on earth would have believed, entirely’: 20: 99). And adversity or punishment in this life may be not just punishment, but may lead to an accelerated progression to paradise and, as in Christianity, a test of faith: mundane illness may be because He has something greater in store (Conrad 1999). Prayers are future directed, not to change the past (but forgiveness may be sought for past sins). And submission to God seems a more prominent motif in Islamic than Christian or Judaic theology.^11^

In practice, it is a good idea to separate out such ‘theological fatalism’ from ‘empirical fatalism’ (Acevedo 2008) which has been said to be more common in peasant societies (Wolf 1966).^12^ Does such normative doctrine actually affect individual understanding and action in everyday life? In a questionnaire study of attitudes, Acevedo (2008) finds that theological fatalism (e.g. ‘No matter which I want to do or be, there is a much more superior power that fully determines the course of my life’) is certainly greater among Muslims than Christians in two societies where the religions coexist, Lebanon and Indonesia, but empirical fatalism (‘How strongly do you feel that you are in control of what you would like to do in your life?’) is no greater among Muslims than Christians in both these countries: indeed in Indonesia, Christians are more empirically fatalistic than Muslims. Acevedo finds that empirical fatalism is more tied to social position than to religious membership, but there is some statistical association between the two types of fatalism. Anthropologists would of course have numerous objections to this sort of study^13^ but we think it not without interest.

And is the frequent solicitation of Allah (*inshallah*) then, though obviously theologically derived, no more than a customary polite self-abnegation or basically ‘meaningless’ polite phrase?^14^ Maybe, but the longer and more poignant requests and thanks to Allah in these interview excerpts do suggest something more than vapid convention. And yet at the same time it is ambiguous whether these always represented direct requests to God for immediate action rather than pious hopes or sturdy acquiescence.

To make some sense of this, it can be compared to something apparently rather different: deflecting accusations of possessing evil eye. Thirty years ago, the first author (RL) took his six-month old daughter with him for his first fieldwork in Trinidad. They were the only whites along the northern coast, and his blonde blue-eyed daughter was an object of curiosity for the local villagers and then for the religious cult they stayed with. Children scarcely bigger than her would attempt to pick her up and carry her around the wooden huts as a sort of trophy. And elderly matrons would come up to her and say: ‘Oh, isn't she an ugly little thing’, and cheerfully pinch her ear until she cried. Now in spite of the wonderful Trinidad tradition of *picong* or barbed satire, RL took this as far from malicious. (He did receive plenty of actual semi-malicious picong himself.) And he saw similar actions directed towards others in the village: nobody would say ‘What magnificent mangoes your tree has!’ or ‘I do admire your new boat’ – or ‘your garden’, or ‘wife’, or ‘child’. Such a statement would have led to suspicions of envy and, if something adverse happened, to assumptions (seldom accusations) of evil eye (*maljo).^15^* One never went out of one's way to praise anything, but one might grudgingly acknowledge some merits or even better say the obvious inverse. ‘An ugly little thing’ meant ‘a pretty little thing’, and making her cry was a guarantee that nothing worse would befall her. And more recent fieldwork in Albania and the Lebanon has yielded a remarkably similar pattern of evil eye avoidance as in the West Indies (Littlewood 2007).^16^ (There are some minor differences: in Albania, for instance, evil eye is only operative before midday.) Anything growing or being built is liable to evil eye: in Trinidad, RL's host family's chicken enclosure was protected by a blue bottle (a sort of general all-purpose banishment of evil and anti-malevolence device), and in Albania houses being constructed are protected with suspended dolls or images with large eyes to deflect the evil eye. Similar blue eyes as apotropaic pendants or brooches may be seen in Greek or Turkish grocer's shops in London.

The evil eye is unconscious in all these (and other) countries and as such is perhaps a way of refusing conscious responsibility for others’ misfortune. To quote Ibn Khaldun (2005, 396): ‘A person who kills by means of sorcery or a miraculous act must be killed, but a person who kills with the eye must not be killed.’ You don't wish to do it or know that you are doing it but certain people do have a reputation for it. So one has to take precautions to make sure you are not doing it or to make sure that nobody suspects you. On the rare occasion of having done it, the correct course is to say you had not intended it and to apologise (not an easy thing if the animal or child has died). Compare this with Evans-Pritchard's (1937) rather similar interpretation of Zande witchcraft.^17^ The evidence of evil eye is that the growing organism or building is withered (*blighted* in Trinidad English), and will fade away and die. The finer points of curing blight from evil eye by secret Latin prayers in both countries are beyond detailed consideration here, except to note that in Trinidad (and perhaps once in Albania) evil eye can almost – almost – be motivated. *Bad talking* (gossip) and *long eye* (envy) in Trinidad similarly shade into frank *dirty ways* (the euphemism for sorcery), with the underlying motive being the same: a desire to gain or destroy at the expense of others, to covet their wealth, or plant, or child, or youth, or beauty, such that you assimilate it to yourself and leave the other diminished and bereft. And in the borderlands between the motivated and the unconscious, RL found the same thing with zombification accusations in Haiti, as Ardener has in Cameroon, and express envy is of course the standard rationale behind sorcery (Douglas 1970).

Fatalism is the flip side to egalitarianism and communal solidarity. The authors would suggest, quite simply, that in small-scale face-to-face communities, such as rural Trinidad or Bangladesh (and still among the socially constricted Bangladeshi communities in London), it is just not appropriate to attribute too much to personal agency or even to be aware of one's own agency: the overtly ambitious or too confident person is an object of suspicion and fear. ‘The over-eager [Muslim] peasant moves ahead of the collective rhythms which assign each act its particular moment in the space of the day, the year or human life; his race with time threatens to drag the whole group into the escalation of diabolic ambition … And thus to turn circular time into linear time, simple reproduction into indefinite accumulation’ (Bourdieu 1977, 162). In what Levi-Strauss (1962) called ‘cold societies’, with relatively little social change or mobility such as, in our case, archaic Arabia, late medieval Europe, or post-colonial village Bengal and Trinidad, local social relations follow a ‘zero sum’ mentality: if you are getting wealthy it is at my expense, your success is my failure.^18^ As Ibn Khaldun (2005, 297) puts it, ‘Whatever is obtained by one is denied to the other, unless he gives something in exchange.’ If you are so sure of your success, how come? What unhallowed things have you got working for you? Have you forgotten your obligations to your fellows so soon? Do you really think you can do it on your own, you who think yourself so self-sufficient? And in situations of potential advancement (Islamic expansion, early modern Europe, contemporary modernity,^19^ one has to be careful to displace any assumptions of self-advancement or well-being outside the customary limits of community obligations to avoid arousing jealous accusations and resentment, and thus losing social and cultural support.

Too facile optimism can be suspect and it is better to attribute major successes and misfortunes to some inscrutable deity or providence. Effortless agency and positive thinking, self-sufficiency and confident health, all can be dangerous. In the words of Samuel Beckett, ‘Do not despair, one of the thieves was saved; do not presume, one of the thieves was damned.’

## Notes

1. As Gellner (1992) has noted, purist ‘High’ Islam (literate, rule-abiding, scriptualist, puritanical, clerical, unitarian) has, in the modern world with urbanisation and modern information technologies, infiltrated and is replacing ‘Low’ Islam (peasant, popular, syncretic, magical, ecstatic). See Husain's (2007) autobiographical account for Bangladeshis in England.

2. *jahu* and *korni:* both (morally neutral) magic and particularly malevolent sorcery (sometimes called ‘black magic’ by Bangladeshis in England).

3. Amulet containing Qur'anic verses or other mantra used for sorcery as well as (more commonly) healing.

4. ‘*Ufri* [evil airs] is when you walk underneath a tree maybe and something catches you like say bad air, you can get bad air from the graveyard too. [Some informants said it could not happen in England, others that it did.] But *korni –* sorcery – is when someone does something to you. Azim's wife Jasmin thought it was korni because Azim's cousin had wanted him to marry her. But Azim's dad didn't want it, and wanted to go up market and get Azim married to Jasmin. Azim was good-looking at the time, you could sell him because of that. Jasmin's relatives were influential, her uncle was a chairman [of a village council], her family were from a higher class than Azim. Azim's family are lower caste.’ Sorcery can happen both in England and (particularly) Bangladesh, or pass from Bangladesh to England.

5. *Shaytaan* (Satan): either the Devil himself, a malevolent non-Muslim jiini or simply ‘the rebellious part of your personality’ [as one informant put it].

6. Spiritually purified by ritual ablutions and prayer.

7. Pir: ‘living saint', a revered religious figure in Bangladesh usually with the power to offer spiritual counselling and healing. Along a spectrum between purist ('Saudi') Islam and the more ecstatic and thaumaturgical, and sometimes seen locally as ‘mad’ (Wilce 2000).

8. Probably an English loan word for jiin.

9. *pora pani:* water over which Qur'anic verses have been recited.

10. Such as the significant ‘Abbasid dispute between the Mu'tazilites and the Ash'arites as to whether (i) mundane causality was illusory, consequent on God creating and sustaining matter only for a successive single moment (Fakhry 2004, 54–56), and (ii) whether the Qur'an itself was created or uncreated (and thus as to whether God's unity was compromised). As opposed to the Mu'tazites, the Ash'arites argued that whilst God certainly had foreknowledge of human action this could be accommodated to God's omniscience as He allows people to ‘acquire’ actions that were created by Him at the moment of action thus preserving human agency (Ruthuen 1997, 58–59, Campanini 2008, 6–9, 51–153, Fakhry 2004, 41–54). Some Muslim sects such as the Khardarites continued to deny predestination until quite late in the history of Islam (see Ibn Battuta 2009, 86). And the Shites were generally less enthusiastic (Fakhry 2004). Ibn Sīnā (Avicenna) rejected the notion of chance or natural determinism by stating that based upon the Qur'an, ‘God's providence means not only that He is the ultimate cause of everything but also responsible for the concatenation of causes’ (Belo 2007: 125). He goes as far as to say that man's attempt to influence God's will through praying is redundant since even man's action of praying has been envisaged by God and embedded into the greater determination (Belo 2007, 129). Ibn Khaldūn (2005, 11) holds that communities when growing in population and wealth become weak and lethargic, and consequently are an easy prey for other less developed communities. A nation collapses as it attains a certain level, in favour of an upcoming nation which in turn will later collapse when it attains a certain level of development. This natural law, used to explain the social and political fragility of the Maghreb in the fourteenth century CE, is based upon the assumption that this historic determination is unchangeable as it is God's will (Mojuetan 1981, 100). Nor, as contemporary anthropology, is this paper directly concerned with the recent philosophical debates (Holton 2010, Mele 2010) following Benjamin Libet's famous experiments as to whether humans actually do have agency – active free will – but rather with the social meaning of what people think, of whether they experience themselves as possessing agency, and under what limits they feel they initiate actions: in other words, the indigenous psychology of agency, agency as a mode of thought. This has recently become of interest in medical social science in arguing whether ‘biological predestination’ (e.g. in genetic screening) has any influence on whether people perceive an illness as determined and thus as uncontrollable (Senior, Marteau, and Peters 1999). And thus, whether ultimate biological or spiritual causation leads to greater ‘fatalism’ about the illness (and thence to less stigmatisation: Littlewood, Jadhav, and Ryder 2007).

11. *Islam* means surrender of the self (to God). Jews are famous for arguing with God: almost unthinkable in Islam.

12. The authors could of course argue religious fatalism elsewhere in small-scale communities (Young et al. 2011).

13. That is, the relevance of a questionnaire approach in these two countries; the local meaning of the questionnaire's wording; the political and social context of each group in the particular country; literacy; context of the interview; origin and deportment of the interviewer, and so on.

14. RL's father, a classically educated Quaker agnostic, would occasionally add onto such parting expressions as ‘See you then next week’ a brief ‘DV’ (*deo volente*, God willing) with perhaps an air of concern for your wellbeing or perhaps as a simple *jeu d'esprit.* The authors are not convinced he was engaging in divine solicitation, maybe wryly referring to the unpredictability of human plans ('careful how you go') and directed to some generalised sense of an unpredictable fate but not to a personalised divinity.

15. *maljo:* from Spanish *mal ojo,* evil eye (Littlewood 2007).

16. And as *nozor* in Bangladesh itself [Callan 2005, Gardner 1997], although none of the informants in the current study mentioned it. It is countered with a black dot painted on the child's forehead.

17. The authors would venture that evil eye passed with the Spaniards from the Mediterranean to the Spanish Main and the Caribbean, then absorbing similar African ideas of witchcraft from among the slaves.

18. Compare ‘hot’ societies: change and mobility, the lauding of the entrepreneur and ‘trickle down’ economics, Adam Smith's invisible hand, with individual greed generating general prosperity.

19. ‘Zero sum’ community solidarity is obviously not restricted to the small scale ‘tribal’ societies to which Levi-Strauss was referring (Geschiere 1997, Wolfe 1966). And with greater opportunities for advancement in England, Bangladeshis there may experience a (temporary) heightened sense of betrayal, not least in their relations with Sylhet (Gardner 1995). (Although, of course, relative to White Britons, Bangladeshis have limited opportunities for economic development and self-assertion [Gardner 2002].) In contemporary post-colonial Trinidad, this presumed egalitarianism is demonstrated by a sentiment of ‘crab antics’ (once caught, a crab can safely be left in an open barrel with a load of other crabs, for any crab trying to climb out will be pulled down by the others; a single crab left to itself will easily get out), articulated by a whole local repertoire of *picong:* ‘The higher a monkey climb the more he expose himself (Littlewood 1993). Residues of the same egalitarian impulse may be found in modern Britain, particularly among the ‘old’ working class who modestly deprecate our relative wealth or achievements – as if boasting of them will bring down the wrath of Fate or Deity upon us (cf. tempting fate; hubris and nemesis; fingers crossed; gambling wins are not ‘real money’; ‘let me die then but please not yet’, and so on). As if one cannot quite believe in one's success and that it will be swept away: perhaps the origins of this lie in humanity's evolutionary past in resenting the free rider. And as the authors’ colleague Yemi Oloyede has pointed out, in West Africa, rationalisations of the denial of relative good health or wealth has the immediate practical advantage of avoiding unwelcome requests for loans, gifts and assistance. A poor Bangladeshi will say the rich were given wealth by Allah as a test of their generosity (Gardner 1995, 146). For Kabyle: ‘Lord, give to me that I may give’ (Bourdieu 1977, 239). Pierre Bourdieu 1977, 224) notes that in contemporary (1950s) Algeria, Kabyle avoid measuring anything for even that is too close to limiting, breaking, cutting your life: a preference for ambiguity means the downplaying of agency. ‘Nothing is more foreign than the attempt to secure a hold over the future, and nothing is more strange to it than the idea of an immense and open future as a broad field of innumerable possibilities which man is able to explore and dominate’ (Bourdieu 1977, 56).
